# A rare case of primary signet‐ring adenocarcinoma of anorectal region in a young patient: Diagnostic challenges and therapeutic outcomes

**DOI:** 10.1002/ccr3.9422

**Published:** 2024-09-08

**Authors:** Bisma Shaikh, Areeba Gul, Ajeet Singh, Hamza Irfan, Tooba Ali, Riyan Karamat, Aymar Akilimali

**Affiliations:** ^1^ Department of Internal Medicine Jinnah Sindh Medical University Karachi Pakistan; ^2^ Department of Internal Medicine Dow University of Health Sciences Karachi Pakistan; ^3^ Department of Medicine Shaikh Khalifa Bin Zayed Al Nahyan Medical and Dental College Lahore Pakistan; ^4^ Department of Internal Medicine Rahbar Medical and Dental College Lahore Pakistan; ^5^ Faculty of Medicine Official University of Bukavu Bukavu Democratic Republic of Congo

**Keywords:** abdominoperineal resection, anorectal adenocarcinoma, multimodal treatment approach, primary signet‐ring cell carcinoma

## Abstract

**Key Clinical Message:**

Primary signet‐ring cell carcinoma of the anal canal and rectum is an extremely rare and aggressive malignancy. The present case underscores the importance of considering primary signet‐ring cell carcinoma in differential diagnoses for young patients with chronic anorectal symptoms. It highlights the need for a multidisciplinary treatment approach (including surgery, chemotherapy, and radiotherapy) and comprehensive follow‐up for managing this challenging condition and improving long‐term patient outcomes.

**Abstract:**

Primary signet‐ring cell carcinoma of the anal canal and rectum is an exceedingly rare subtype of colorectal adenocarcinoma, often originating as an extension of rectal adenocarcinoma. This malignancy constitutes a small fraction of colorectal cancers and is scarcely reported in medical literature. We present the case of an 18‐year‐old male with a three‐year history of progressively worsening hematochezia, anorectal pain, and defecation‐associated prolapse. Initial conservative treatments failed, leading to further investigations that revealed a palpable, nodular anorectal mass. Imaging studies (including CT and MRI), and biopsy confirmed poorly differentiated adenocarcinoma with signet‐ring cell morphology. The tumor exhibited extensive lymphovascular invasion and involved perirectal lymph nodes, and was staged as pT3, N2a. Immunohistochemical staining was positive for CK 7, CK 20, and SATB2, supporting the primary anorectal origin. The treatment regimen included initial diversion colostomies for symptom relief, followed by neoadjuvant chemotherapy with a modified 5‐fluorouracil, leucovorin, irinotecan, and oxaliplatin (FOLFIRINOX) regimen and concurrent chemoradiation with Xeloda. The patient subsequently underwent an abdominoperineal resection (APR), which confirmed the diagnosis and achieved curative resection. Postoperative complications included transient ileus and wound infection, which were managed with supportive care. This case underscores the diagnostic and therapeutic challenges posed by primary signet‐ring cell carcinoma of the anorectal region, highlighting the need for a high index of suspicion and comprehensive diagnostic workup in atypical presentations. The multimodal treatment approach, incorporating surgery, chemotherapy, and radiotherapy, was crucial in managing this locally advanced tumor. The rarity and aggressiveness of this carcinoma necessitate a tailored treatment strategy to improve patient outcomes. Long‐term follow‐up, including regular imaging and surveillance, is vital for monitoring disease recurrence and evaluating treatment effectiveness.

## 1 INTRODUCTION

Primary signet‐ring cell carcinoma of the anal canal and rectum is an infrequent condition which predominantly arises from an extension of a rectal adenocarcinoma. This form of adenocarcinoma is rare when compared with other forms of adenocarcinoma of the large intestine, and is rarely discussed in the existing global medical literature.^1^, ^2^, ^3^ Because of the infrequency with which this cancer is encountered, there is little reliable information concerning differences between standard colorectal adenocarcinoma and the signet‐ring cell variant. It initially manifests with anal pain, rectal bleeding, and perianal mass.^3^ In adolescents and young adults, 0.8% of colorectal cancers are found to exhibit a signet cell carcinoma.^4^ At least 50% of the cells being examined should exhibit signet cell caricature to be categorized as a signet cell carcinoma, as per the classifications of the WHO. Direct transpapillary cold forceps biopsy under fluoroscopy can be used for signet‐ring cell carcinoma diagnoses. This method allows for precise sampling of the tumor tissue, ensuring an accurate diagnosis and deciding the treatment strategy.^5^ Current clinical practice guidelines recommend a similar surgical treatment approach for anorectal adenocarcinomas as for rectal neoplasms.^6^ Although surgery remains the common course of treatment for early‐stage malignancies, simultaneous chemoradiation therapy along with a subsequent resection is a favorable treatment option in the case of locally advanced tumors.^6^ Abel et al.'s study comprising 77% of patients undergoing resection for the treatment of anal adenocarcinoma, demonstrated that surgical treatment via an abdominoperineal resection (APR) shows favorable patient outcomes.^7^ The present case aims to highlight a unique occurrence of poorly differentiated, primary signet‐ring cell adenocarcinoma involving both the anal canal and rectum. This case report has been reported in line with the SCARE Criteria.^8^

## 2 CLINICAL PRESENTATION

### 2.1 Case history/examination

An 18‐year‐old Pakistani male presented with a 3‐year history of progressively worsening hematochezia, anorectal pain, and prolapse on defecation. Initially attributed to benign causes such as hemorrhoids, conservative measures, including dietary modification and topical therapies, were attempted without significant improvement. Throughout this period, the patient experienced increasing discomfort and distress due to persistent symptoms, which significantly impacted his quality of life and daily activities. Despite these efforts, the patient's condition continued to deteriorate, leading to weight loss, fatigue, and decreased appetite. This ongoing decline and lack of response to initial treatments ultimately prompted a referral to a colorectal specialist for further evaluation. Upon detailed history‐taking, the patient reported no significant past medical history or family history of gastrointestinal malignancies; yet, it should be noted that his father and a maternal uncle were both diagnosed with lung cancer, while his mother's cause of death was intracranial bleeding (ICB). The complete hematological and biochemical profile of the patient is shown in Table 1. Furthermore, physical examination revealed a palpable mass in the anorectal region, accompanied by tenderness and ulceration. A digital rectal examination demonstrated a firm, nodular mass involving the rectal wall with evidence of bleeding on manipulation. There were no palpable inguinal lymph nodes, and the systemic examination was otherwise unremarkable.

**TABLE 1:** Hematological and biochemical profile of the patient.

Test	Result	Reference range
Blood urea nitrogen	24 mg/dL	6–20
Creatinine	1.4 mg/dL	Females: 0.6–1.1; Males: 0.9–1.3
Sodium	131 meq/L	136–146
Potassium	3.4 meq/L	3.5–5.1
Chloride	98 meq/L	98–106
Hemoglobin	10 gm/dL	Adult male 13–18; Adult female 11.5–16
RBC count	3.5 × 10^6^mL/μL	Adult male 4.5–5.8; Adult female 3.7–5.1
HCT	38%	Adult male 45–58; Adult female 37–50
MCV	77.2 fL	Adult 76–96
MCH	29.8 pg	Adult 28–32
MCHC	34.6 g/dL	Adult 32–36
Total leukocytes count	10.1 × 10^3^/μL	Adult: 4.0–11.0
Neutrophils	78%	Adult 50–75
Lymphocytes	18%	Adult 20–50
Eosinophils	3%	Adult 1–6
Monocytes	5%	Adult 1–6
Basophils	0.7%	Adult 0–1
Platelet count	198 × 10^3^/μL	150–400
RDW‐SD	53 fL	‐
RDW‐CV	16.1%	‐

Abbreviations: HCT, hematocrithaematocrit; MCH, mean corpuscular hemoglobin; MCHC, mean corpuscular hemoglobin concentration; MCV, mean corpuscular volume; RBC, red blood cell; RDW‐CV, red cell distribution width‐coefficient of variation; RDW‐SD, red cell distribution width‐standard deviation.

### 2.2 Differential diagnosis

Given the atypical presentation and lack of response to conservative measures, a broad differential diagnosis was considered, including inflammatory bowel disease, infectious colitis, anal fissures, and malignant etiologies such as anorectal carcinoma and melanoma. Diagnostic uncertainty warranted further investigations to definitively delineate the underlying pathology.

### 2.3 Investigations

**FIGURE 1:**
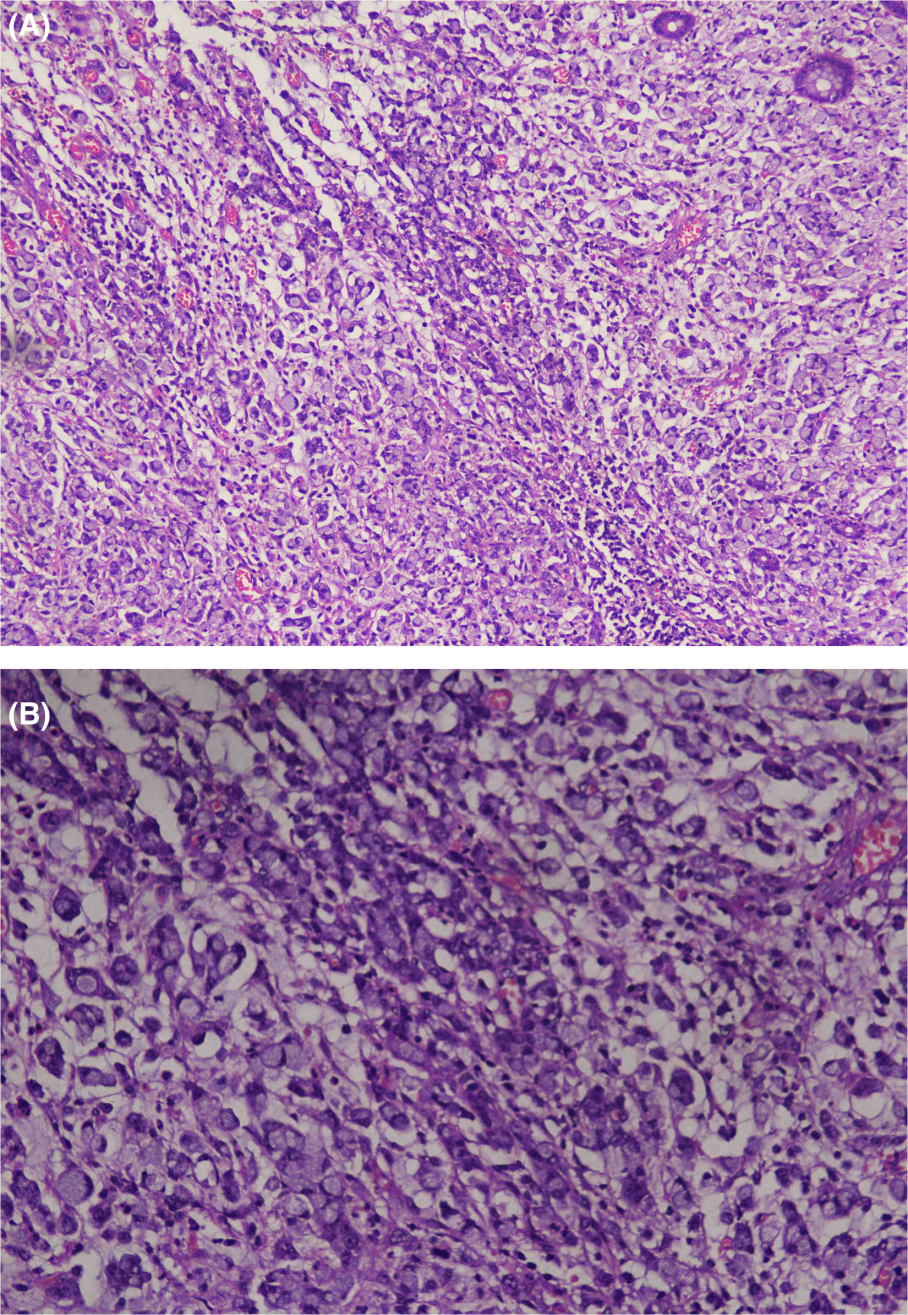
(A, B) Histopathological features consistent with poorly differentiated adenocarcinoma, with signet ring cell morphology.

A CT scan revealed circumferential minimally enhancing wall thickening in the anorectal region, with subsequent findings of the collapsed sigmoid colon and neoplastic mass. Biopsy specimens obtained during endoscopy demonstrated histopathological features consistent with poorly differentiated adenocarcinoma, with signet ring cell morphology noted on microscopy, supporting the diagnosis of a rare anorectal adenocarcinoma (Figure 1). Post‐colostomy surgery and post‐chemo/radiotherapy CT scans revealed a collapsed sigmoid colon, thickening of the distal rectum, and anal canal with a neoplastic mass. The wall thickness at the 6 o'clock position measured approximately 1.3 cm. MRI showed no enhancement of the lesion for recurrent or residual disease. The carcinoembryonic antigen (CEA) test showed a positive result of 24.34 ng/dL (*N* < 5.00 ng/dL). There was no evidence of any adrenal, bony metastatic, hepatic, or pleuropulmonary deposits. Moderate left‐side pleural effusion was spotted, caused by infection or inflammation.

**FIGURE 2:**
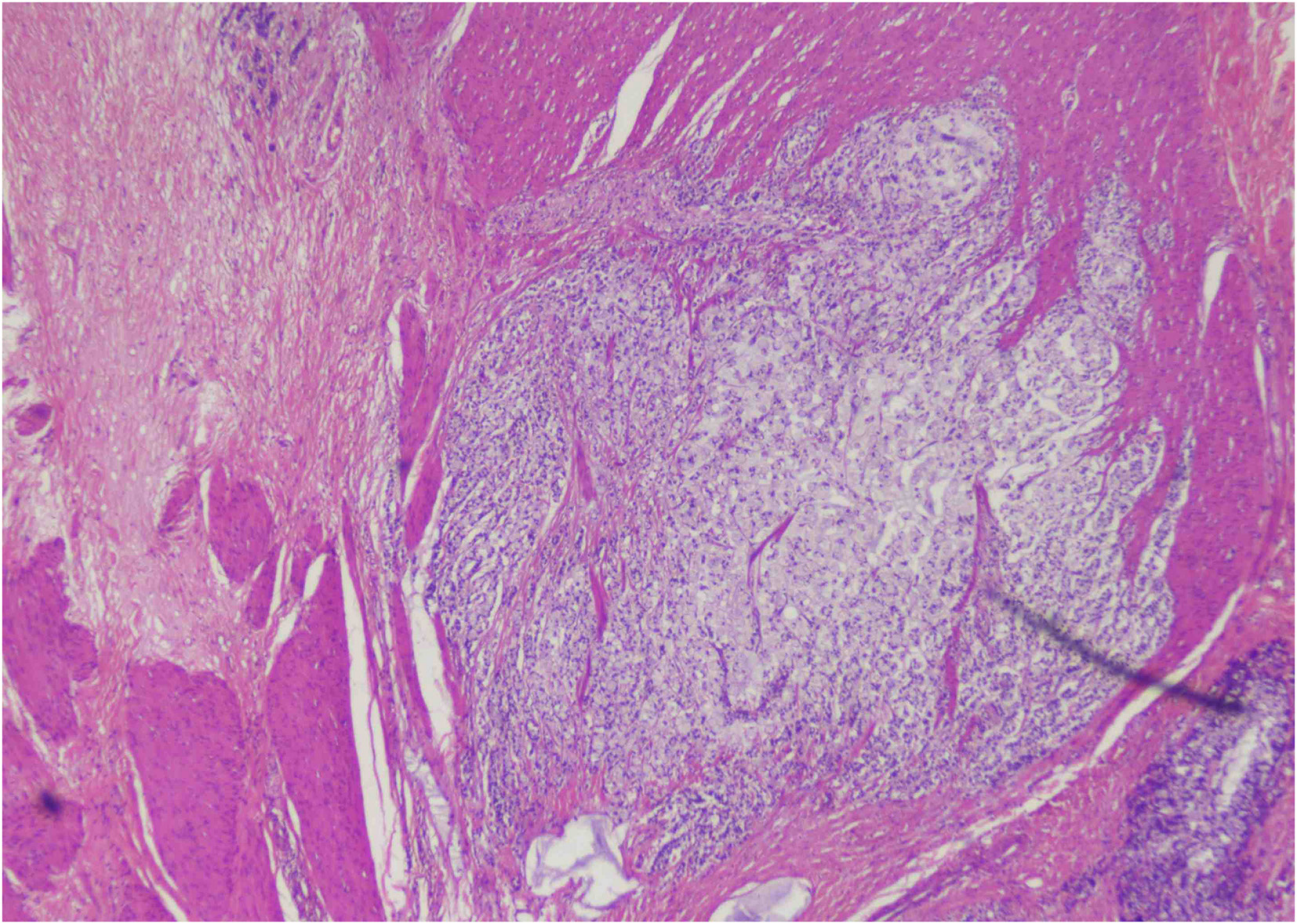
Histopathological section showing anorectal signet ring carcinoma invading the muscularis propria.

**FIGURE 3:**
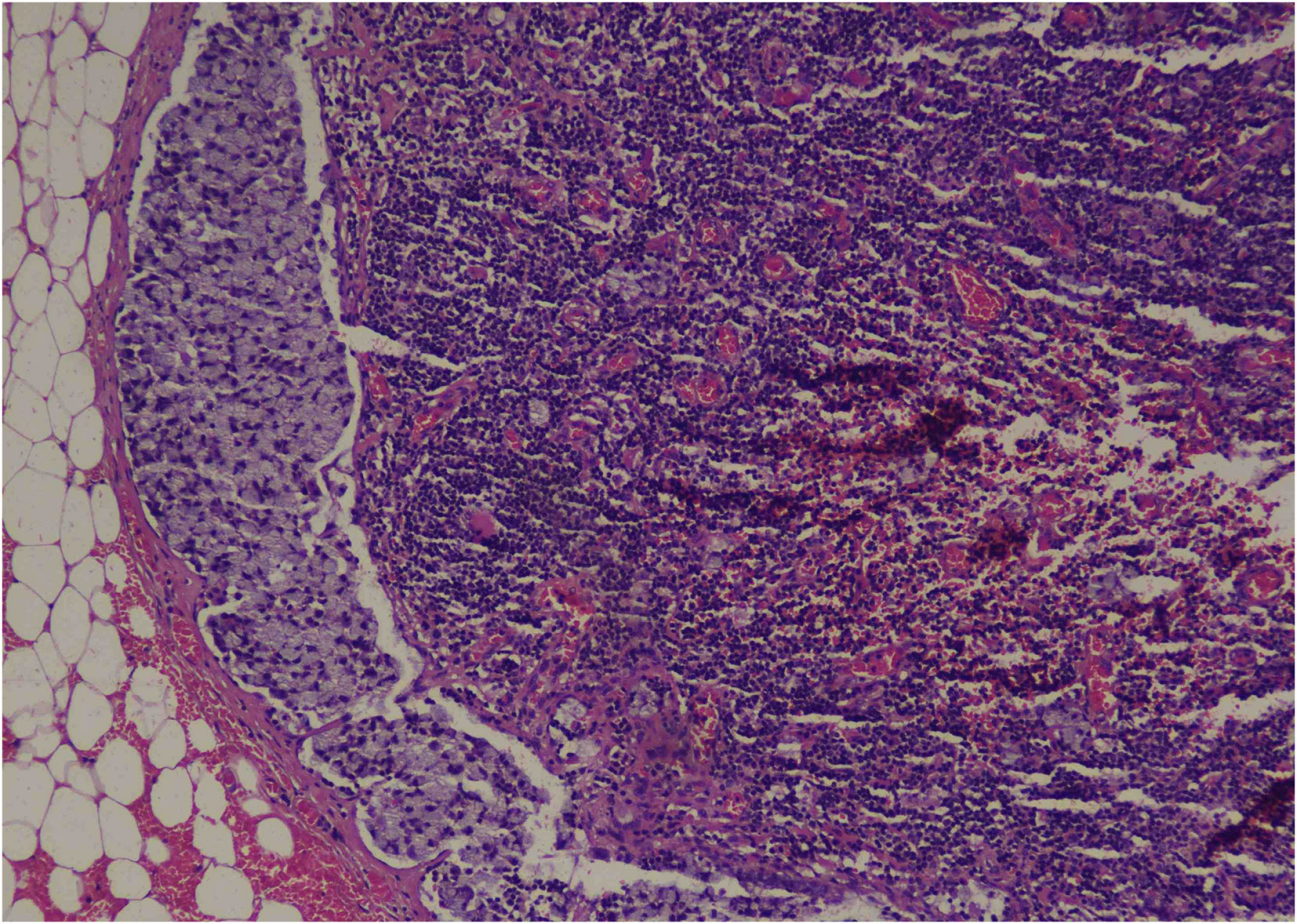
Biopsy findings illustrate the infiltration of carcinoma in lymph node parenchyma.

**FIGURE 4:**
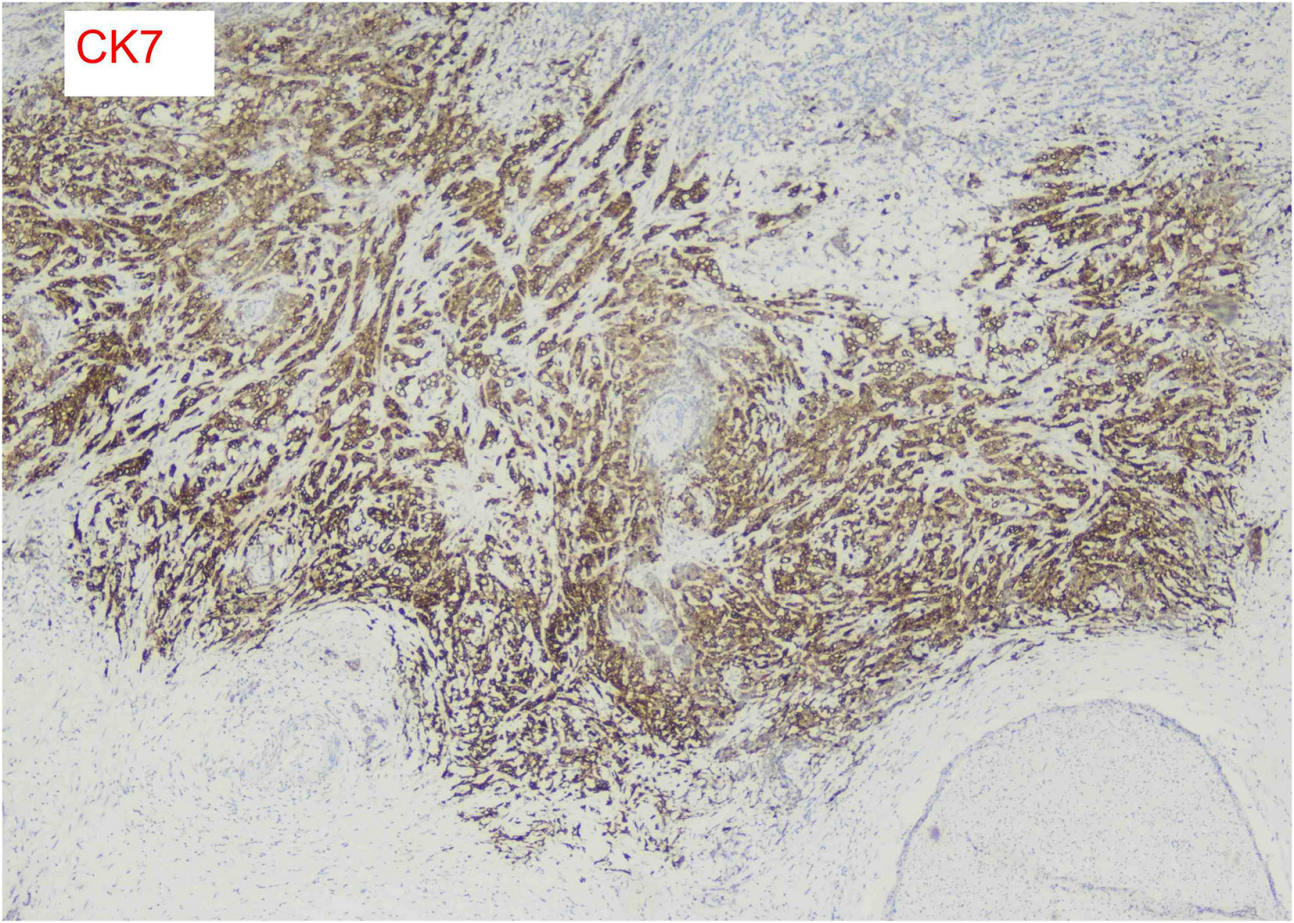
The immunohistochemical stains show the cytokeratin positivity of tumor cells (Tumor cells were focally positive for CK 7 and diffusely positive for CK 20).

In early February 2024, the patient underwent APR for a permanent colostomy, pain reduction, and a definitive diagnosis to guide further management. It was revealed that the tumor site was located in the rectum, below the peritoneal reflection, and involving the anal canal. The pathologic stage of the tumor was pT3, N2a. The tumor exhibited a fibrotic configuration and extended through the muscularis propria (Figure 2) into the perirectal fat, with extensive lymphatic and vascular invasion being extensively present (Figure 3). Multiple tumor deposits (discontinuous extramural extension) were observed, while perineural invasion was not identified and macroscopic tumor perforation was not present. Microscopic assessment showed complete intactness of the mesorectum. A total of 16 lymph nodes were recovered, with 5 involved by the tumor. Furthermore, immunohistochemistry for MLH1, MSH2, MSH6, and PMS2 showed intact expression, in addition to positive SATB2, a novel biomarker. The immunohistochemical stains showed that the tumor cells were focally positive for CK 7 and diffusely positive for CK 20 (Figure 4). The tumor size was measured at 3 × 2 × 1.6 cm. In relation to the peripheral margins, the tumor was 20 cm away from the proximal margin, 2.5 cm away from the distal margin, and the circumferential margin was involved by the tumor. There was no detection of polyps, diverticula, ulcers, or strictures. Furthermore, the stoma was identified at a distance of 2.6 cm from the proximal resection margin and measured 2.5 × 2.4 cm, with the stoma site being tumor‐free. These detailed pathological findings provided crucial information for further treatment planning and prognostication.

### 2.4 Treatment

The patient underwent initial management with diversion colostomies to alleviate obstructive symptoms and optimize nutritional status. Following the operation, chemotherapy was initiated after 2 weeks. The chemotherapy regimen consisted of six cycles administered over a span of 6 months, while radiotherapy comprised 28 cycles delivered over 2 months. The patient underwent six cycles of the modified 5‐fluorouracil, leucovorin, irinotecan, and oxaliplatin (FOLFIRINOX) regimen. Subsequently, concurrent chemoradiation therapy with Xeloda was administered after completing these initial six cycles. This was followed by adjuvant chemotherapy with the mFolfox 6 protocol.

Subsequent restaging evaluations demonstrated a favorable response to neoadjuvant therapy, with a reduction in tumor size and the absence of distant metastases. The patient subsequently underwent APR with en bloc resection of involved pelvic structures, including the rectum, anus, and regional lymph nodes. Postoperatively, blood transfusion was administered due to low hemoglobin and hematocrit levels, and serum potassium abnormalities were corrected with KCl administration.

**FIGURE 5:**
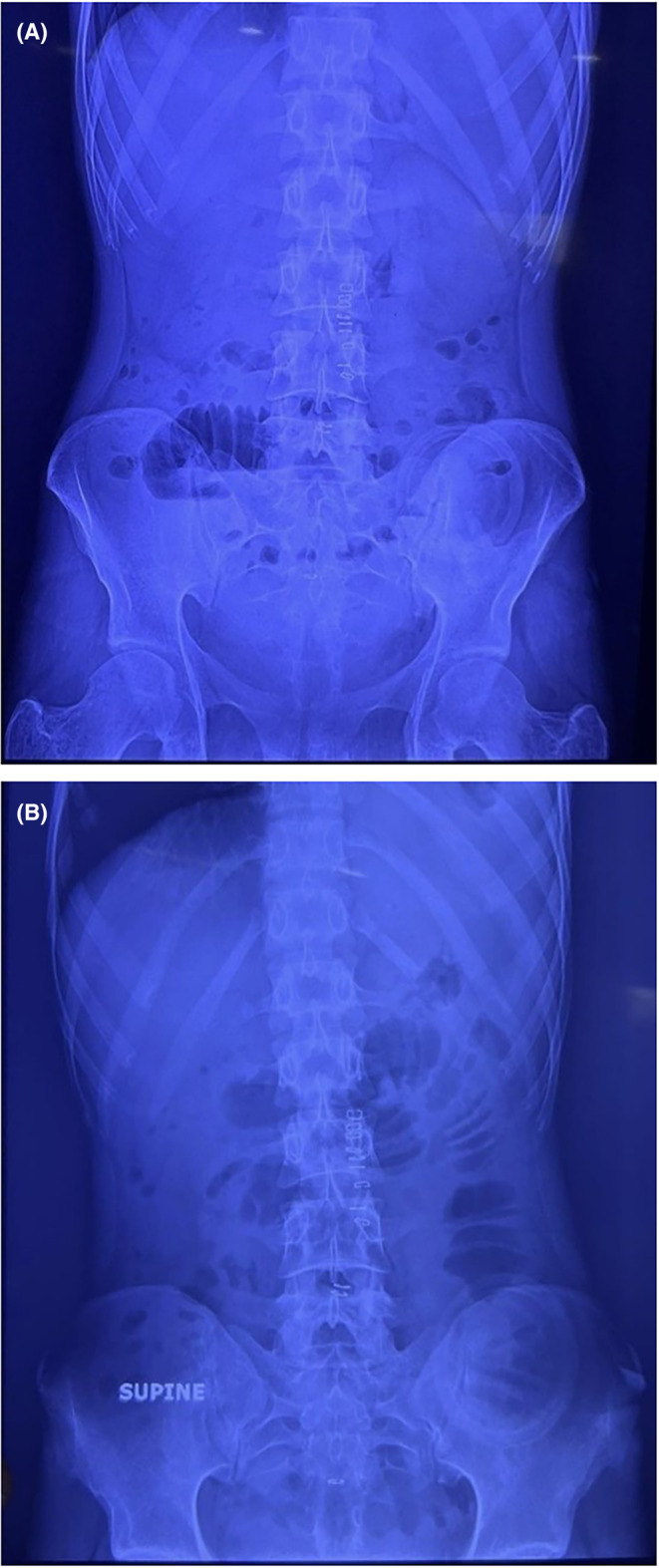
(A) x‐ray illustrating the presence of air‐fluid levels. (B) x‐ray indicating small bowel obstruction.

The patient presented post‐APR with vomiting and a non‐functioning stoma, indicating small bowel obstruction. X‐rays confirmed the obstruction, and lab investigations revealed low serum potassium, chloride, albumin, and creatinine levels, remedied by KCl administration based on the patient's weight. Subsequent x‐ray results exhibited air‐fluid levels (Figure 5A) and small bowel obstruction (Figure 5B).

### 2.5 Postoperative management

Adjuvant chemotherapy with a modified FOLFOX regimen was initiated postoperatively, aiming to eradicate residual disease and reduce the risk of locoregional recurrence. However, postoperative recovery was complicated by transient ileus, wound infection, and stoma dysfunction, necessitating close monitoring and supportive care. Ileus, a temporary cessation of bowel function, was managed conservatively with bowel rest, nasogastric decompression, and gradual reintroduction of oral intake. The wound infection required antibiotic therapy and local wound care. These complications were transient and resolved with appropriate management. The postoperative ileus and wound infection had a notable impact on the patient's recovery. Ileus prolonged the hospital stay and delayed the initiation of postoperative adjuvant therapy, which is critical in preventing recurrence. The wound infection contributed to increased discomfort and required additional medical interventions, potentially affecting the patient's overall quality of life during the recovery period.

### 2.6 Follow‐up

Despite these challenges, the patient gradually improved, with follow‐up evaluations showing no evidence of disease recurrence at 4 months post‐surgery. However, the psychological and physical impact of a permanent colostomy significantly affected the patient's quality of life, necessitating ongoing support and counseling. Long‐term follow‐up, including serial imaging studies and surveillance colonoscopies, remains integral to monitoring for disease recurrence and evaluating treatment efficacy. The patient continues to undergo regular clinical assessments and oncological surveillance, with ongoing multidisciplinary management aimed at optimizing long‐term outcomes and quality of life.

## 3 DISCUSSION

We provide a unique case of primary signet‐ring cell carcinoma involving anorectal region in a young adolescent male presenting with a 3‐year duration of anorectal pain, prolapse on defecation as well as hematochezia. Investigations confirmed anorectal adenocarcinoma. The treatment included diversion colostomies, neo‐adjuvant therapy, and abdominoperineal resection. The postoperative complications were as follows: a brief ileus and small bowel obstruction. Neoadjuvant chemotherapy was carried out to decrease the risk of recurrence. This case is an example of the diagnostic and therapeutic problems to be faced in an anorectal adenocarcinoma in a young person. Despite the atypical presentation and the absence of typical conservative management, a careful diagnostic workup revealed a definite diagnosis.

Anorectal adenocarcinoma is a kind of rare colorectal cancer subtype that is characterized by the mucin presence within the tumor cells and is among the less frequent subtypes, accounting for up to 1% of all registered colorectal malignancies.^9^ It is also worth noting that this malignancy poses significant challenges to both diagnosis and treatment. Certain variants of this form, for instance, mucinous adenocarcinoma associated with the anorectal fistula, display reasonably unique features, such as aberrant fluorodeoxyglucose (FDG) accumulation, which can be detected by FDG‐PET/CT and differentiates them from mucinous adenocarcinomas occurring in colorectal cancer.^10^ Besides these, anorectal adenocarcinomas are also associated with other conditions, such as perianal Paget's disease, in which the disease is not limited only to the perianal skin.^11^ In terms of histology, anorectal adenocarcinomas display a range of subtypes, namely, mucinous adenocarcinomas, tubular adenocarcinomas, and squamous cell carcinomas.^12^ The range of histological entities extends from squamous cell carcinoma, adenocarcinoma, melanoma, gastrointestinal stromal tumors, poorly differentiated neuroendocrine tumors, and lymphoma.

Another important finding was the fact that some of the anorectal adenocarcinomas expressed p16, which could be associated with human papillomavirus.^1^ It is crucial to differentiate primary anorectal melanoma from anorectal adenocarcinoma. Despite its rarity, it still has been recorded.^13^ This type of cancer accounts for a significant portion of colorectal adenocarcinomas and presents unique challenges for its treatment and prognosis. According to The International Agency for Research on Cancer in 2018, the incidence of anorectal tumors reached a crude rate of 25.2 per 100,000 cases annually, and about 187,172 new cases were reported.^14^ Despite extensive research, the correlation between anorectal adenocarcinoma and rectal prolapse remains underexplored. While previous studies have established a connection between chronic rectal prolapse and an increased relative risk of colorectal cancer,^15^ the evidence concerning anorectal adenocarcinoma specifically is close to none and predominantly retrospective in nature. Although the simultaneous occurrence of colorectal polyps and solitary rectal ulcers with rectal prolapse has been documented, instances of anorectal adenocarcinoma along with rectal prolapse are rare. Furthermore, Chanjuan et al. reported a unique case of synchronous primary perianal Paget's disease and rectal adenocarcinoma, a phenomenon previously undescribed. They noted that approximately 70%–80% of such cases arise secondary to invasive carcinomas, which signifies the complexity of these presentations.^16^

Screening and diagnosing anorectal adenocarcinoma requires a multifaceted approach due to its complex clinical presentations. Anorectal adenocarcinoma can be linked to other conditions like IBS and primary and secondary diseases. One such case was reported by Kodama et al., where the authors explored the differences between adenocarcinoma associated with Crohn's disease and the more common type found within anorectal fistulae.^17^ Moreover, Liao et al. investigated the use of immunohistochemical stains to differentiate between primary and secondary Paget's disease associated with anorectal adenocarcinoma. Their study demonstrated the use of staining patterns of CK7, GCDFP‐15, CK20, MUC2, and CDX2 to help distinguish Paget's disease from anorectal adenocarcinoma.^18^ In a case report by Demirel et al., the authors explored the diagnostic utility of anorectal smear in identifying anorectal adenocarcinoma. The report discussed a specific case where a tumor was laparoscopically resected which was subsequently confirmed as adenocarcinoma. This case underscores the critical role of diagnostic procedures in confirming the presence of such malignancies.^19^ In another case report, Varma et al. reported a patient with cutaneous metastasis originating from anorectal adenocarcinoma. With the anorectal adenocarcinoma itself being a rare occurrence, its cutaneous metastatic potential highlights the importance of biopsy confirmation of cutaneous lesions.^20^ Such confirmation can significantly influence the treatment approach and prognosis for these patients.

The treatment of anorectal adenocarcinoma is usually based on multimodal approaches, which are outlined in guidelines, including the NCCN. These protocols highlight the importance of proper patient selection for curative treatments, which frequently involve a combination of chemotherapy, radiation, and surgical resection for localized disease.^6^ Furthermore, mitomycin‐based chemoradiation protocols, such as Nigro's protocol for squamous cancer of the anal canal, have been demonstrated to be effective as a primary therapy for anal adenocarcinoma, emphasizing the central role of chemoradiation in the treatment of anorectal adenocarcinoma.^21^ For patients presenting with inguinal lymph node metastases, systemic chemotherapy and radiotherapy should be preferred due to the frequent occurrence of distant metastasis and poor prognosis.^22^ However, in solitary lymph node metastasis, lymph node removal for improved results is recommended.

In our patient's case, the initial treatment consisted of the creation of diversion colostomies to relieve obstructive signs and improve nutritional status. Following this, the patient underwent a modified FOLFIRINOX regimen followed by concurrent chemoradiation therapy with Xeloda. The rationale for selecting the modified FOLFIRINOX regimen was based on its demonstrated efficacy in treating aggressive and advanced‐stage gastrointestinal cancers. It is known for its potent anti‐tumor activity and has shown improved survival rates in pancreatic and colorectal cancers.^23^ Given the patient's advanced disease stage and poor differentiation of the tumor, this aggressive chemotherapy regimen was chosen to maximize tumor shrinkage and control metastatic spread. Concurrent chemoradiation with Xeloda (capecitabine) was selected due to its radiosensitizing properties, which enhance the effectiveness of radiotherapy in targeting residual tumor cells and minimizing locoregional recurrence.^24^ Alternative treatment options, such as single‐agent chemotherapy or less intensive regimens, were considered but deemed less suitable due to the high risk of disease progression and the need for a more aggressive approach to manage this rare and aggressive cancer subtype. The selected treatment protocol aimed to achieve the best possible outcome by addressing both the local and systemic components of the disease, thereby improving the patient's chances for long‐term survival and disease control.

After a successful APR and response to neoadjuvant therapy, postoperative complications developed including temporary ileus, wound infection, and stoma dysfunction. Moreover, preoperative radiotherapy has been proven to be effective, mainly in the management of locally advanced perianal mucinous adenocarcinoma, a sign that it can be useful in the selected cases of anorectal adenocarcinoma.^25^ It should be emphasized that as a result of the rapid progress in treatment modalities, anorectal adenocarcinoma can present challenges, especially in cases of spread. Long‐term follow‐up, including periodic imaging studies and surveillance colonoscopies, plays a key role in recurrence and efficacy assessment.

## 4 CONCLUSION

In summary, anorectal adenocarcinoma is a complicated clinical picture that may need a combination of diagnostic measures and widely used treatment methods. Despite our increasing knowledge of the clinicopathological features of gastric cancer and the available therapeutic interventions, this malignancy is still difficult to manage, especially in cases where the presentation is atypical and several comorbidities are involved. Large‐scale studies are needed to investigate the relationships between anorectal adenocarcinoma and different risk factors and to improve diagnostic methods and treatment protocols. Both the increase in data collection and the analysis are important to customize individual approaches for the diagnosis, treatment, and monitoring and, therefore, to improve the outcomes for this rare but clinically significant disease.

## AUTHOR CONTRIBUTIONS

**Bisma Shaikh:** Conceptualization; data curation; project administration; supervision; validation; visualization; writing – original draft; writing – review and editing. **Areeba Gul:** Project administration; supervision; validation; visualization; writing – original draft; writing – review and editing. **Ajeet Singh:** Project administration; supervision; validation; visualization; writing – original draft; writing – review and editing. **Hamza Irfan:** Validation; visualization; writing – original draft; writing – review and editing. **Tooba Ali:** Validation; visualization; writing – original draft; writing – review and editing. **Riyan Karamat:** Validation; visualization; writing – original draft; writing – review and editing. **Aymar Akilimali:** Validation; visualization; writing – original draft; writing – review and editing.

## FUNDING INFORMATION

The authors did not receive any funding for this work.

## CONFLICT OF INTEREST STATEMENT

The authors declare no conflicts of interest.

## ETHICS STATEMENT

Ethical approval was not required for this case report.

## CONSENT

Written informed consent was obtained from the patient to publish this report in accordance with the journal's patient consent policy.

## Data Availability

Data sharing not applicable to this article as no datasets were generated or analyzed during the current study.
